# Bone mineral density predictors in long-standing type 1 and type 2 diabetes mellitus

**DOI:** 10.1186/s12902-021-00815-5

**Published:** 2021-08-06

**Authors:** Stefana Catalina Bilha, Letitia Leustean, Cristina Preda, Dumitru D. Branisteanu, Laura Mihalache, Maria-Christina Ungureanu

**Affiliations:** 1 Endocrinology Department, “St. Spiridon” Emergency Hospital, “Grigore T. Popa” University of Medicine and Pharmacy, No. 16 University Street, 700115 Iasi, Romania; 2 Department of Diabetes Mellitus, Nutrition and Metabolic Diseases, “St. Spiridon” Emergency Hospital, “Grigore T. Popa” University of Medicine and Pharmacy, No. 16 University Street, 700115 Iasi, Romania

**Keywords:** Diabetes mellitus, Bone mineral density, HbA1c

## Abstract

**Background:**

Despite the increased fracture risk, bone mineral density (BMD) is variable in type 1 (T1D) and type 2 (T2D) diabetes mellitus. We aimed at comparing independent BMD predictors in T1D, T2D and control subjects, respectively.

**Methods:**

Cross-sectional case-control study enrolling 30 T1D, 39 T2D and 69 age, sex and body mass index (BMI) – matched controls that underwent clinical examination, dual-energy X-ray absorptiometry (BMD at the lumbar spine and femoral neck) and serum determination of HbA1c and parameters of calcium and phosphate metabolism.

**Results:**

T2D patients had similar BMD compared to T1D individuals (after adjusting for age, BMI and disease duration) and to matched controls, respectively. In multiple regression analysis, diabetes duration – but not HbA1c- negatively predicted femoral neck BMD in T1D (β= -0.39, p = 0.014), while BMI was a positive predictor for lumbar spine (β = 0.46, p = 0.006) and femoral neck BMD (β = 0.44, p = 0.007) in T2D, besides gender influence. Age negatively predicted BMD in controls, but not in patients with diabetes.

**Conclusions:**

Long-standing diabetes and female gender particularly increase the risk for low bone mass in T1D. An increased body weight partially hinders BMD loss in T2D. The impact of age appears to be surpassed by that of other bone regulating factors in both T1D and T2D patients.

## Background

Diabetes mellitus is a chronic whole-body disease leading to a wide range of complications, such as cardiovascular disease, retinopathy, nephropathy, neuropathy and also “sweet bone” disease [1]. Although the underlying pathophysiological background is very different, type 1 (T1D) and type 2 diabetes (T2D) are both associated with an increased fracture risk - which is multifactorial and only partially explained by falls and bone mineral density (BMD) [2]. The most consistent effect is upon the hip fracture risk, ranging between 2.4- and 7-fold increase in T1D [3] and being two to three times higher in T2D compared to the general population [1].

Diabetic osteopathy in T1D and T2D is characterized by low serum vitamin D, negative calcium balance, low bone turnover and high sclerostin levels [4]. However, bone mass may differ to some extent in T1D when compared to T2D [5], but not in all studies [6]. Low BMD occurs early after disease onset due to the deleterious effects of insulinopenia upon bone turnover and bone mass accrual in T1D, remaining rather stable afterwards [7]. Reported BMD in T2D varies from unaltered bone density [8, 9] to a paradoxically higher BMD [5] compared to controls. Low bone mass was also found in the later stages of T2D, possibly linked to microvascular disease [10].

Skeletal fragility is nevertheless described in both T1D and T2D, independently of BMD [2]. Advanced glycation end products (AGEs) alter the structure of the collagen, promote oxidative stress and inflammation, and also contribute to low bone turnover [1, 3]. The effect of glycemic control - reflected by HbA1c levels - upon bone is inconsistent, with some studies reporting an elevated fracture risk with increasing HbA1c [11, 12], while bone density evolution appears rather independent of HbA1c levels [5, 6]. In T2D, the protective effect of an increased body weight and hyperinsulinemia upon bone are counterbalanced by the negative impact of increased visceral adiposity and insulin resistance, an inadequate adaptation of bone strength to increased mechanical load, the long duration of disease evolution and various anti-diabetic drugs (e.g., thiazolidinediones or sodium-glucose cotransporter type 2 inhibitors – SGLT-2) [1].

We aimed at investigating independent predictors of BMD in T1D and T2D patients compared to controls with regard to general and diabetes - specific parameters.

## Methods

### Study design and subjects

Patients diagnosed with diabetes (T1D and T2D) were consecutively recruited during routine follow-up visits for disease monitoring in the Diabetes, Nutrition and Metabolic Diseases Clinic of “Sf. Spiridon” Clinical Emergency Hospital Iasi (Romania) between January and December 2017. Patients aged between 18 and 80 years old were included if they had a well-established diagnosis of diabetes according to standardized criteria [13] in their original medical record (HbA1c > 6.5 % on two separate tests; T1D: new-onset hyperglycemia accompanied by ketonuria at debut, low serum levels of insulin and peptide C and requiring insulin treatment for control and survival - antibodies to glutamic acid decarboxylase were also tested where the clinical phenotype was rather non-specific, such as slow onset of symptoms, BMI ≥ 25 kg/m^2^ or age over 40 with normal BMI and requiring insulin treatment from the time of diagnosis [14]; T2D: two fasting blood sugar levels ≥ 126 mg/dl or an oral glucose tolerance test showing serum glucose ≥ 200 mg/dl accompanied by a phenotype of insulin resistance and not requiring insulin), were more than 1 year after disease onset, were receiving antidiabetic treatment (without any changes in medication type in the past six months), were at their first bone evaluation, and had an estimated glomerular filtration rate (eGFR) ≥ 60 ml/min/1.73 m^2^ (serum creatinine was measured and eGFR was calculated using the CKD-EPI equation). Age, sex and body mass index (BMI) 1:1 matched apparently healthy volunteer controls (CTL) referred by the general practitioner to the outpatient department in our hospital for general investigations were enrolled in the same period as the patients. Exclusion criteria for both groups were represented by calcium and vitamin D supplementation, bone active therapy (antiresorptive/bone-forming therapy), liver disease, moderate and severe chronic kidney disease (CKD; stage G3 to end-stage renal disease), history of parathyroid or rheumatological disease, oral corticosteroid use > 5 mg prednisone equivalent in the past 3 months or endogenous hypercortisolism, hypo- and hyperthyroidism, inflammatory bowel disease, hypogonadism (other than menopause), smoking (both regular and heavy) and heavy drinking (more than 2 drinks per day or more than 15 drinks per week for men and more than 1 drink per day or more than 8 drinks per week for women). Subjects exhibiting hyperglycemia (an abnormal fasting blood sugar, impaired glucose tolerance or diabetes) were further excluded from the CTL group.

Sixty-nine patients (30 T1D and 39 T2D) and 69 age, sex and BMI-matched CTL that were willing to participate and met the study inclusion and exclusion criteria were recruited in the Diabetes and Endocrinology outpatient clinics, after giving written informed consent and were enrolled in this cross-sectional case-control study. The study adhered to the Declaration of Helsinki and was approved by the institutional Ethics Committee.

### Evaluation and measurements

Complete medical history (anamnesis and medical charts) was recorded for all patients and CTL. The presence of microvascular complications was defined as: [1] nephropathy: positive albumin:creatinine ratio (≥ 30 mg/g) on two or more occasions, [2] retinopathy: positive ophthalmologic fundus examination, [3] polineuropathy: clinical measurement of vibration. Macrovascular complications were defined based on the recorded history of coronary heart disease, stroke, myocardial infarction, or peripheral vascular disease, respectively.

After clinical examination (height and weight were recorded and BMI was calculated as weight (kg) divided by square height (m)), all patients underwent dual-energy X-ray absorptiometry (DXA; Hologic Delphi A, software version 12.7.3.2 Hologic Inc., USA) scanning to measure BMD at the lumbar spine and hip (femoral neck was reported due to lower values compared to total hip, according to the recommendations of the ISCD [15]). Coefficient of variation was 0.39 % for lumbar anterior-posterior spine and 1 % for femoral neck BMD. Measurements were made by two trained technicians certified by the International Society for Clinical Densitometry (ISCD), according to standard protocol and with daily calibration. Least significant change (LSC) was 0.008 g/cm^2^ for lumbar BMD and 0.0104 g/cm2 for femoral neck BMD, respectively. According to the Adult Official Positions of the ISCD [15], T-scores were reported for postmenopausal women and men ≥ 50 years of age (“low bone mass” was defined as T-score <-1 in this category) while Z-scores were recorded for premenopausal women and men < 50 years (“low bone mass” was defined as Z-score ≤-2). Also, if there was a more than 1.0 T-score difference between adjacent vertebrae, the questioned vertebra was excluded from the analysis, while the BMD of the remaining vertebrae was used to derive T-score [15]. Menopause was defined as more than 12 months since natural cessation of menstrual cycles.

On the same day as the clinical and DXA examinations, blood samples were collected after overnight fasting in all study participants. Biochemical analysis of standard clinical parameters included HbA1c determination (ion-exchange high-performance liquid chromatography (HPLC) method), serum calcium and phosphate (colorimetry; Cobas 6000 analyzer, Roche), serum thyroid stimulating hormone (TSH) and parathyroid hormone (PTH) (intact PTH second-generation chemiluminescent enzyme immunometric assay; Immulite 2000 Immunoassay System, Siemens).

### Statistical analysis

SPSS (SPSS Statistics version 20.0 for Windows) was employed for statistical analysis. Data are expressed as mean ± SEM (standard error of the mean). Comparisons between groups (T1D versus T2D, T1D versus controls and T2D versus controls, respectively) were made using Student’s t-test (for normally distributed data) or the non-parametric Mann-Whitney U test (for skewed data), after checking for normal distribution (Shapiro-Wilk test). Analysis of variance (ANOVA) was employed for comparisons between 3 or more categories. The analysis of covariance (ANCOVA) was used to calculate age, BMI and diabetes duration - adjusted BMD values in T1D compared to T2D (least square means ± standard error are reported). Multiple regression analysis was performed to assess independent predictors of bone mass in T1D, T2D and matched-CTL, respectively, as follows: continuous variables potentially influencing BMD variation, such as age, diabetes duration, HbA1c, BMI and PTH, as well as categorical variables (introduced after binary coding 0/1), such as gender (male = 0, female = 1) were introduced as independent variables in regression models with lumbar BMD and femoral neck BMD as the outcome variables, respectively. The level of significance was established for p-value < 0.05.

## Results

Despite being younger and having a mean BMI within the normal reference range, T1D patients had a longer duration of diabetes and a poor glycemic control with more diabetes complications compared to the older, rather obese but with improved glycemic control T2D patients (Table 1). Serum calcium, phosphate, PTH and thyroid status were similar between T1D and T2D patients, although serum PTH had the tendency to be higher in T2D subjects (Table 1). All T1D patients were receiving exogenous insulin treatment, while all T2D patients were under metformin treatment: 16 were following metformin monotherapy, 13 were taking metformin together with a sulfonylurea drug and 10 associated incretin therapy to metformin (none were using thiazolidinediones or sodium-glucose co-transporter-2 inhibitors).

**Table 1:** Characteristics of the study patients

	Type 1diabetes mellitus(*n* = 30)	Control group(*n* = 30)	Type 2diabetes mellitus(*n* = 39)	Controlgroup(*n* = 39)	Type 1vs.Type 2	Type 1vs.Control	Type 2vs.Control
	mean ± SEM		mean ± SEM	mean ± SEM		*p*-value	
**Gender (n)**Women(Menopause)Men	*n* = 18 (60 %)(*n* = 6)*n* = 12 (40 %)	*n* = 18 (60 %)(*n* = 6)*n* = 12 (40 %)	*n* = 19 (48.7 %)(*n* = 16)*n* = 20 (51.3 %)	*n* = 19 (48.7 %)(*n* = 16)*n* = 20 (51.3 %)	--	--	--
**Age (y)**	40.27 ± 2.7	42.4 ± 3.12	62.39 ± 1.21	60.03 ± 1.31	< 0.001	0.61	0.2
**BMI (g/cm2)**	24.27 ± 0.81	25.66 ± 0.73	30.56 ± 0.78	30.77 ± 0.78	< 0.001	0.22	0.84
**Diabetes-specific parameters**							
Duration of diabetes (y)	14.23 ± 1.89	-	9.49 ± 0.8	-	0.026	-	-
HbA1c (%)	8.86 ± 0.35	-	6.86 ± 0.15	-	< 0.001	-	-
Complications (n)	12 (40 %)		9 (23 %)				
**General parameters**							
Calcium (mg/dl)	9.63 ± 0.7	-	9.69 ± 0.6	-	0.49	-	-
Phosphate (mg/dl)	3.43 ± 0.12		3.25 ± 0.08		0.19		
TSH (uUI/ml)	3.3 ± 0.8	-	2.23 ± 0.013	-	0.14	-	-
PTH (pg/ml)	37.98 ± 2.24	37.11 ± 2.33	47.47 ± 3.9	48.42 ± 1.6	0.055	0.79	0.84

*BMI* body mass index, *PTH* parathyroid hormone, *SEM* standard error of the mean, *TSH* thyroid stimulating hormone

BMD values at the lumbar spine and femoral neck were similar between T1D and T2D patients, and also between T1D patients and controls and between T2D patients and controls, in the whole group and according to sex, respectively (Table 2). After adjusting for age, BMI and disease duration, BMD did not vary significantly between T1D and T2D patients, respectively (Table 2). However, fewer patients in the T2D group exhibited low bone mass compared to matched controls, while the number of low bone mass subjects was similar in T1D patients and matched controls (Table 2).

**Table 2:** BMD values in diabetes patients and controls

	Type 1diabetes mellitus	Controlgroup	Type 2diabetes mellitus	Controlgroup	Type 1vs.Type 2	Type 1vs.Control	Type 2vs.Control
	mean ± SEM	mean ± SEM	mean ± SEM	mean ± SEM	*p*-value		
**BMD / Women**	***N* = 18**	***N* = 18**	***N* = 19**	***N* = 19**			
Lumbar BMD(g/cm2)	0.97 ± 0.02	0.97 ± 0.03	0.93 ± 0.04	0.93 ± 0.03	0.47	0.99	0.9
Lumbar T/Z-score	-0.8 ± 0.2	-0.7 ± 0.2	-1 ± 0.3	-1.1 ± 0.3	0.59	0.83	0.91
Femoral neck BMD (g/cm2)	0.74 ± 0.02	0.78 ± 0.03	0.77 ± 0.03	0.76 ± 0.03	0.52	0.29	0.98
Femoral neck T/Z-score	-1.1 ± 0.2	-0.6 ± 0.3	-0.6 ± 0.3	-0.8 ± 0.2	0.21	0.16	0.79
**BMD / Men**	***N *= 12**	***N* = 12**	***N* = 20**	***N* = 20**			
Lumbar BMD(g/cm2)	1.09 ± 0.03	1 ± 0.05	1.08 ± 0.04	1.03 ± 0.03	0.91	0.15	0.3
Lumbar T/Z-score	-0.2 ± 0.3	-0.8 ± 0.5	-0.1 ± 0.3	-0.6 ± 0.3	0.76	0.26	0.3
Femoral neck BMD (g/cm2)	0.94 ± 0.03	0.9 ± 0.04	0.9 ± 0.04	0.92 ± 0.03	0.38	0.48	0.52
Femoral neck T/Z-score	0.1 ± 0.3	-0.2 ± 0.4	-0.3 ± 0.3	0 ± 0.2	0.38	0.55	0.42
**BMD/Total**	***N* = 30**	***N* = 30**	***N* = 39**	***N* = 39**			
Lumbar BMD(g/cm2)	1.01 ± 0.02	0.98 ± 0.03	1.01 ± 0.03	0.98 ± 0.02	0.88	0.3	0.89
Lumbar T/Z-score	-0.6 ± 0.2	-0.8 ± 0.2	-0.6 ± 0.3	-0.8 ± 0.2	0.85	0.5	0.94
Femoral neck BMD (g/cm2)	0.82 ± 0.03	0.83 ± 0.28	0.83 ± 0.03	0.85 ± 0.02	0.79	0.81	0.79
Femoral neck T/Z-score	-0.6 ± 0.2	-0.4 ± 0.21	-0.5 ± 0.2	-0.4 ± 0.2	0.61	0.53	0.55
Adjusted BMD* (LSMEAN ± SE)	***N* = 30**	***N* = 30**	***N* = 39**	***N* = 39**			
Lumbar BMD (g/cm2)	1.04 ± 0.04	-	0.99 ± 0.03	-	0.46	-	-
Femoral neck BMD (g/cm2)	0.84 ± 0.04	-	0.82 ± 0.03	-	0.7	-	-
**WHO-criteria**							
Low bone mass	N = 8	N = 8	N = 13	N = 17			

*adjusted for age, body mass index and diabetes duration

*BMD* bone mineral density, *LSMEAN* least square mean, *SE* standard error, *SEM* standard error of the mean, *WHO* world health organization

### BMD predictors in T1D, T2D and controls

General (age, gender, BMI and PTH) and diabetes - specific parameters (disease duration, HbA1c) were introduced as independent variables in multiple regression analysis with lumbar and femoral neck BMD as outcome variables, respectively (Table 3). Gender independently predicted BMD across all models: compared to men, female sex was an independent risk factor for low BMD in T1D, T2D patients and controls, respectively. In T1D patients, diabetes duration was also a negative independent predictor of femoral neck BMD, while BMI was a positive independent predictor of both lumbar and femoral neck BMD in the T2D group - but not in controls. Age was a negative independent predictor of BMD in controls (both T1D and T2D controls), but not in patients with diabetes (Table 3).

**Table 3:** Multiple regression analysis of BMD predictors in patients with type 1 and type 2 diabetes mellitus and controls, respectively

Model	Dependent variable	T1D model	T2 model	Predictor	T1D		T2D	
					Beta	p-value	Beta	p-value
1	Lumbar BMD	*R*^2^ = 0.43 *P* = 0.038	*R*^2^ = 0.37 *P* = 0.014	Age Gender Diabetes duration HbA1c BMI PTH	0.15 ***-0.66*** -0.36 0.07 -0.3 0.27	0.5 ***0.002*** 0.075 0.7 0.16 0.21	-0.03 ***-0.52*** 0.16 0.02 ***0.46*** -0.1	0.87 ***0.002*** 0.33 0.89 ***0.006*** 0.51
2	Femoral Neck BMD	*R*^2^ = 0.68 *P* < 0.001	*R*^2^ = 0.42 *P* = 0.006	Age Gender Diabetes duration HbA1c BMI PTH	-0.08 ***-0.66*** ***-0.39*** 0.13 0.12 0.08	0.64 ***< 0.001*** ***0.014*** 0.33 0.45 0.63	-0.26 ***-0.55*** 0.27 -0.11 ***0.44*** 0.09	0.11 ***0.001*** 0.09 0.46 ***0.007*** 0.55
**Model**	**Dependent variable**	**Control T1D model**	**Control T2 model**	**Predictor**	**Control T1D**		**Control T2D**	
					Beta	p-value	Beta	p-value
1	Lumbar BMD	*R*^2^ = 0.27 *P* = 0.036	*R*^2^ = 0.32 *P* = 0.01	Age Gender BMI PTH	***-0.45*** ***-0.31*** 0.04 0.05	***0.011*** ***0.05*** 0.81 0.75	***-0.41*** ***-0.45*** 0.17 -0.17	***0.013*** ***0.005*** 0.36 0.33
2	Femoral Neck BMD	*R*^2^ = 0.35 *P* = 0.006	*R*^2^ = 0.51 *P* < 0.001	Age Gender BMI PTH	***-0.31*** ***-0.5*** 0.04 -0.06	***0.05*** ***0.002*** 0.81 0.69	***-0.39*** ***-0.67*** 0.15 -0.23	***0.006*** ***< 0.001*** 0.35 0.12

*BMD* bone mineral density, *BMI* body mass index, *PTH* parathyroid hormone, *T1D* type 1 diabetes, *T2D* type 2 diabetes

### Subgroup analysis

#### The presence of diabetic complications

**Fig. 1:**
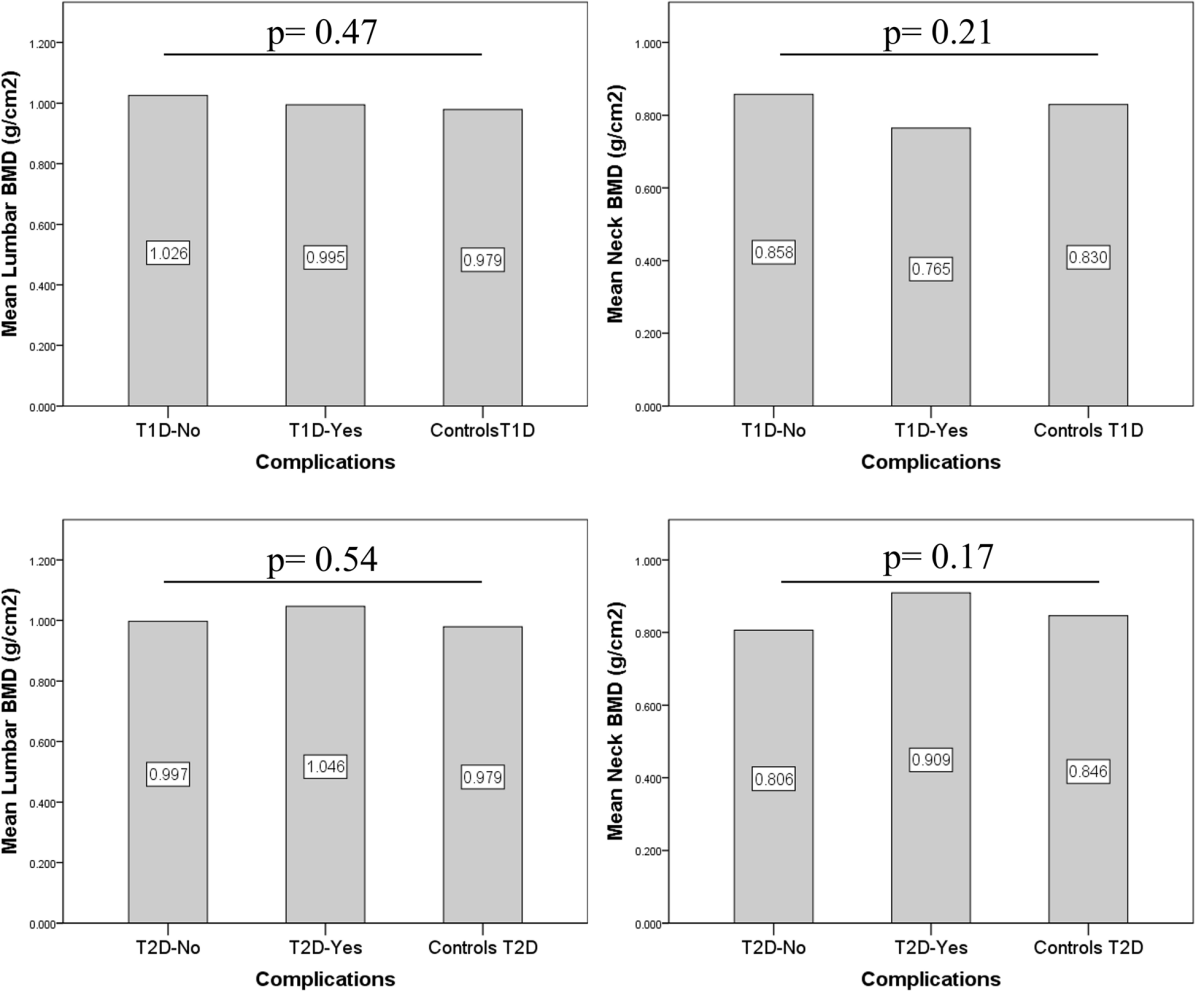
BMD values according to the presence of complications in type 1 and type 2 diabetes patients compared to controls. BMD = bone mineral density, T1D = type 1 diabetes mellitus, T2D = type 2 diabetes mellitus. T1D patients with complications – *n* = 12, T1D patients without complications – *n* = 18, T2D patients with complications – *n* = 9, T2D patients without complications – *n* = 30, T1D controls – *n* = 30, T2D controls – *n* = 39

T1D patients with diabetes complications (n = 12) had similar lumbar BMD values compared to T1D patients without complications (n = 18) and controls (n = 30), respectively (p = 0.47); however, they tended to have a rather lower femoral neck BMD, but the differences did not reach statistical significance (0.764 g/cm^2^ in T1D patients with complications versus 0.858 g/cm^2^ in T1D patients without complications versus 0.829 g/cm^2^ in matched controls, respectively, p = 0.21). More so, T1D patients with diabetes complications had longer disease duration (20.5 ± 3.3 versus 10.1 ± 1.68 years, *p* = 0.012) compared to T1D patients without complications, despite similar HbA1c and BMI (data not shown). BMD did not differ significantly between T2D patients with (n = 9) and without (n = 30) complications and controls (n = 39), respectively (Fig. 1).

#### Drugs in T2D

In the T2D group, we compared BMD across different treatment categories. Although patients taking metformin + sulfonylurea tended to have a rather lower bone density compared to the other subgroups, lumbar BMD (1.03 ± 0.05 versus 0.95 ± 0.05 versus 1.04 ± 0.04, *p* = 0.42) and femoral neck BMD (0.84 ± 0.04 versus 0.8 ± 0.05 versus 0.86 ± 0.04, respectively) did not differ significantly among metformin monotherapy (*n* = 16), metformin + sulfonylurea (*n* = 13) and metformin + incretin therapy (*n* = 10), respectively.

## Discussion

T1D and T2D patients had similar BMD compared to controls, respectively. Diabetes duration, but not HbA1c, was found to negatively predict femoral neck BMD in T1D, but not T2D patients. In the T2D group, BMI was an independent predictor of bone density, while female gender was negatively associated with low BMD in both T1D and T2D, independently of other factors. Unlike patients with diabetes, age was the other major independent predictor of bone mass in controls, in addition to gender.

Albeit BMD underestimates fracture risk in patients with diabetes, it still remains the cornerstone in bone evaluation in this particular group due to high accessibility and low costs [16]. Data reporting BMD values in T1D and T2D patients are very heterogenous and rather inconsistent with regard to BMD predictive factors.

An older meta-analysis [5] reported negative Z-scores for T1D patients and positive Z-scores for T2D subjects, thus concluding that T1D is associated with lower bone mass, while T2D patients generally have higher BMD. More recent studies reported, however, similar BMD values both between patients with diabetes and controls and between T1D and T2D patients, respectively [6, 17]. Another recent study performed investigating bone mass in long-standing (longer than 50 years) T1D patients with good glycemic control and low rates of vascular complications reported similar or even better BMD expressed as Z-score compared to age-, gender- and race-matched population [18]. We found lower bone mass scores at the femoral neck in T1D women compared to T2D women and controls; nevertheless, the differences did not reach statistical significance, probably due to the limited number of patients. Also, more women in the T2D group were postmenopausal compared to the T1D group, and this may account for the lack of a statistical difference regarding BMD between types of diabetes. More so, the different stages of evolution and disease management captured in various studies, the potentially erroneous diabetes classification and also the adjustment for various confounding variables may account for the variability of reported data. The early and rather acute insulinopenia associated with diabetes onset impairs bone mass accrual and negatively impacts peak bone mass. Thus, bone mass acquisition is hampered in the early stages of T1D [3]. Nonetheless, bone density was demonstrated to stabilize or even increase after exogenous insulin treatment is well installed, with studies reporting age – and gender - expected bone density measurements [19]. More so, T1D patients with low bone mass are reported to follow lower insulin dose regimens compared to those with normal bone mass [20].

Disease duration - and not age – proved to be one of the main independent predictors of low femoral neck BMD in T1D patients in our study, suggesting that diabetes-related factors, such as diabetes duration, may be more important for bone. Indeed, T1D patients experiencing diabetes-specific complications had a longer disease history and also the tendency towards lower femoral neck BMD. Low rates of vascular complications have been linked to preserved BMD in long-lasting T1D [18]. According to recent consensus in the field, diabetes-specific risk factors for fracture include age, low BMD, the presence of complications of diabetes, disease duration, previous fractures and glycemic control (particularly in T1D with HbA1c > 8–9 %) [21]. Our results are in agreement with other studies reporting long-lasting disease as a risk factor for fragility fractures [11, 22]. The presence of micro- and macrovascular complications is associated with low BMD [5, 6] and was also reported to increase fracture risk [22, 23]. Microvascular complications as a result of collagen glycation and impaired bone turnover due to AGEs are thought to compromise bone quality and material properties, thereby significantly increasing fracture risk [2, 24, 25]. Although we and others [26] failed to find any significant BMD variation according to the presence of complications, microvascular damage is demonstrated to alter bone microarchitecture, possibly via VEGF linking diabetic complications and skeletal health. This explains the disproportionate fracture risk in T1D versus T2D, compared to differences in BMD [27]. Complications are also associated with longer disease history, an independent factor for low BMD, once again supporting the link between bone mass and microarchitecture changes and the long exposure to diabetic milieu. Similar to other studies [6, 20], we failed to find a significant effect of HbA1c (which shows only the severity of recent diabetes dysregulation) upon BMD. However, we did not assess fracture risk, as long standing poor glycemic control is known to be associated with increased fracture risk, independently of bone mass [20].

T2D patients in the current study had similar BMD compared to matched controls, although fewer patients in the diabetes group exhibited low bone mass. T2D patients are generally reported to have increased BMD compared to reference populations, although not in all studies [5, 28]. Potential disease misclassification, lack of a matched control group and inability to adjust for covariates are important sources of bias and heterogeneity [28]. Diabetes duration is an important confounder: the osteoanabolic effects of the hyperinsulinemia secondary to insulin resistance may explain the apparently higher bone density in early T2D, while insulinopenia in T1D and late T2D is accompanied by sarcopenia and low bone mass [1]. Despite using metformin which is known to positively impact bone mass and reduce fracture risk [29, 30], the T2D patients in the current study had a rather long disease history of approximately 10 years, with one quarter also experiencing complications. A diabetes duration longer than 5 years is a risk factor for low bone mass [31] and the presence of microvascular complications in T2D is associated with lower cortical volumetric BMD and altered bone microarchitecture, namely increased cortical porosity and diminished cortical thickness at the radius [32]. BMD did not differ in patients with complicated T2D compared to T2D patients without complications in the current study. At the same time, our T2D patients had a good glycemic control, while an increased HbA1c is associated with increased BMD according to the meta-analysis of Ma et al.[28]. Other meta-analyses failed to find a significant correlation of HbA1c with BMD in T2D [5]. Also, BMD progressively increases with clinical cutoffs for fasting glucose (normal, impaired and overt T2D) [33]. However, this increased BMD may be explained by the diminished bone mineral area of these patients, which also exhibit low bone turnover as assessed by serum markers, such as osteocalcin or cross-laps [33].

An increased BMI is a protective factor against osteoporosis in all populations [34] (including patients with diabetes [34]), via the increased mechanical loading. Obesity is a risk factor for insulin resistance and diabetes [35], being at the same time associated with higher areal and volumetric BMD and improved cortical bone structure [1, 35]. It also contributes to higher BMD in T2D patients, as demonstrated by the current study and also by many others [28]. At the same time, diabetes and obesity are associated with systemic inflammation and adipokine dysregulation, all contributing to impaired bone metabolism [36]. Despite variable BMD, alterations in cortical bone microarchitecture are reported in T2D patients, explaining the higher fracture risk compared to the reference population [37].

None of the patients in our study were under anti-diabetic therapy known to negatively impact bone mass, such as thiazolidinediones or SGLT-2 inhibitors. While all T2D patients were using the “bone-friendly” metformin, the subgroup also using a sulfonylurea drug tended to have a rather lower BMD, without reaching statistical significance. This is still to be clarified as the mechanisms of action of the sulfonylurea class of medication upon bone remain unelucidated up to present [38].

Age and gender (female sex was associated with a lower BMD compared to men, independently of other factors) were the main independent BMD predictors in the reference populations in our study. Interestingly, the effect of age, unanimously recognized as a risk factor for low bone mass, was not detected in the T1D and T2D patients in our study. It is possible that other factors surpass the effect of aging upon BMD in diabetes, particularly in young or obese patients.

Our study is limited by the relatively small number of patients, lack of assessment of bone microarchitecture, bone turnover markers or fracture risk. The effect of vitamin D levels, known to be altered in individuals with diabetes [4], was also not assessed. Nonetheless, the presence of matched control groups for T1D and T2D subjects, respectively, together with the evaluation of BMD predictors in patients with diabetes versus matched healthy individuals are important study strengths.

## Conclusions

Female sex and long-standing diabetes particularly increase the risk for low BMD in T1D, with special concern for the femoral neck. An increased BMI partially contributes to BMD preservation in T2D, independently of age; however, appreciating bone mass to its real extent is rather difficult in T2D due to various contributing factors to bone changes.

## Data Availability

The datasets used and/or analysed during the current study are available from the corresponding author on reasonable request.

## Abbreviations

AGEs: Advanced glycation end products
BMD: Bone mineral density
BMI: Body mass index
CKD: Chronic kidney disease
CTL: Control group
DXA: Dual energy X-ray absorptiometry
eGFR: Estimated glomerular filtration rate
ISCD: International Society for Clinical Densitometry.
PTH: Parathyroid hormone.
SGLT2: Sodium-glucose co-transporter-2.
T1D: Type 1 diabetes mellitus.
T2D: Type 2 diabetes mellitus.
TSH: Thyroid stimulating hormone.
